# Strengthening of 0.18 wt % C Steel by Cold Differential Speed Rolling

**DOI:** 10.3390/ma15103717

**Published:** 2022-05-22

**Authors:** Jee-Hyun Kang, Young-Gun Ko

**Affiliations:** School of Materials Science and Engineering, Institute of Materials Technology, Yeungnam University, Gyeongsan 38541, Korea; jeekang@yu.ac.kr

**Keywords:** 0.18 wt % C steel, differential speed rolling, ultrafine grain, mechanical properties, grain boundary strengthening

## Abstract

Steel sheets containing 0.18 wt % C were deformed by differential speed rolling (DSR) up to four passes and compared to the steel sheets processed by equal speed rolling (ESR). Not only microstructure, but also mechanical properties and rolling load, were studied, which enlightens the relationship between microstructure, mechanical properties, and rolling load. Moreover, microstructure and properties resulting from ESR were systematically compared. During the rolling deformation, coarse grains were elongated first parallel to the rolling direction, and ultrafine grains were subsequently formed via continuous dynamic recrystallization. Microstructural analysis revealed that DSR was more effective than ESR in terms of achieving grain refinement and microstructure homogeneity. High-angle grain boundaries surrounding the ultrafine grains contributed to grain boundary strengthening, resulting in a dramatic increase in both hardness and strength after DSR. Although the steel was strengthened by rolling, the rolling load firstly increased and subsequently decreased as the number of passes increased, and lower force was required during DSR than during ESR. These can be explained by considering deformation volume and sticking friction.

## 1. Introduction

Rolling is the most widely employed metal forming process. Thus, various studies on the rolling process have been reported until now [1,2,3,4,5]. In recent years, differential speed rolling (DSR) has been regarded as one of the asymmetric rolling (ASR) methods in which the metallic or composite sheets were deformed between two distinctive rolls with different dimensions or speeds. As the name implied, DSR utilized the identical rolls driven under different rotational speeds at both high and room temperatures. Because DSR could successfully impose intensive shear strain throughout the thickness of the workpieces [6], many attempts have been made to achieve severe grain refinement in a variety of metallic materials, such as Mg [7], Ti [8], Al [9], and Fe [10] alloys.

An advantage of DSR is that a high degree of shear deformation is produced uniformly throughout the thickness. According to the texture prediction assisted by the finite element method (FEM), better texture was realized by DSR than by single-roll-driven rolling [11]. In addition, the mesh distortion obtained via FEM simulation suggested that the shear displacement was larger in DSR than in equal speed rolling (ESR) [12].

As a result of the effective shear deformation in DSR, ultrafine grain (UFG) structure with a high dislocation density was achieved regardless of the crystal structure of alloys [6]. The UFG structure developed gradually over the course of several rolling passes. At first, the grains elongated parallel to the rolling direction. As the accumulated thickness reduction increased, the UFG structure was generated throughout the thickness. In the case of Al alloys, it was reported that the relatively homogeneous microstructure with 0.7 μm grains was observed after four DSR passes when the roll speed ratio was 4 and the thickness reduction per pass was 30%. Moreover, these fine grains were surrounded by a higher fraction of high-angle grain boundaries (HAGB) than that of low-angle grain boundaries (LAGB) [13]. Indeed, a higher fraction of HAGB appeared in low-carbon steel sheets after DSR than after ESR [14].

The UFG microstructure with HAGB effectively contributed to the materials strength. Therefore, a DSR processed Cu sheet exhibited higher strength than an ESR processed Cu sheet [15]. Furthermore, the strength of the sheets produced by DSR was comparable to that produced by severe plastic deformation such as accumulative roll bonding (ARB) [15] and equal channel angular pressing (ECAP) [6].

Recently, the microstructure of low-carbon steels produced by DSR was studied. Hamad et al. analyzed UFG microstructure with HAGB and texture associated with both rolling and shear deformation after four DSR passes with the roll speed ratio of 4 [16]. Wronski and Bacroix also confirmed the texture with a shear component throughout the thickness after DSR with a roll speed ratio of 1.3 [17]. In addition, Hamad and Ko studied the effect of roll speed ratio and concluded that the fraction of ultrafine grains, as well as strength, increased by increasing the roll speed ratio from 1 to 4 [18]. Moreover, the UFG structure developed during DSR was found to be thermally stable upon annealing up to 798 K (525 °C) for 1 h [19]. Most of these studies focused on microstructure and texture evolution during DSR, and the mechanical properties were rarely studied, along with microstructure analysis.

In this regard, the present study investigated the strengthening of a 0.18 wt % C steel sheet, which was processed by DSR. The microstructure was analyzed, and hardness, as well as tensile properties, were investigated after the DSR process. Moreover, these features were compared with the counterpart produced by ESR to prove the efficacy of DSR on grain refinement and strengthening. Additionally, the rolling load was monitored throughout the process, and the load response was interpreted based on the material strength and deformation volume.

## 2. Materials and Methods

The composition of the steel sheets was Fe-0.18 C-0.5 Mn-0.012 Si-0.007 Cr (wt %). The steel was machined into 100 × 30 × 4.4 mm^3^ sheets. The sheets were homogenized at 1373 K (1100 °C) for 3 h and furnace cooled, which resulted in the mixed microstructure containing ferrite and 20% pearlite. The mean lineal intercept grain size of ferrite was 32 ± 4 µm.

Two identical rolls, each with a 220 mm diameter, were employed for DSR. The roll speed ratio was 4, while the lower roll speed was 5 rotations per minute. Thickness reduction per each pass was ~30%, which resulted in ~75% after 4 passes. The rolling was performed for 1, 2, and 4 passes. Lubrication was provided, and rolling load was monitored throughout the rolling process. Some sheets were also produced by 4 passes of ESR for comparison. All processes were completed at room temperature without rotating the sheets. Details of processing conditions are available elsewhere [16].

The microstructure after DSR was analyzed with a field-emission scanning electron microscope (FE-SEM, Hitachi S-4300, Tokyo, Japan) equipped with electron backscatter diffraction (EBSD). The specimens were cut in the middle of the RD-ND plane, mechanically polished, and etched with 2% picric acid in ethanol. EBSD samples were prepared by mechanical polishing, which was finished with colloidal silica. Ferritic regions were separated as a subset for EBSD analysis, which was performed by TSL OIM 6.1.3 (Mahwah, NJ, US). The step size for the EBSD acquisition was 0.020 µm. For the TEM (Tecnai G2 F20, Hillsboro, OR, US) analysis, thin foils were prepared via focused ion beam (FEI Quanta 3D FEG, Hillsboro, OR, USA).

Nanoindentation (Nanovea Nanoindenter, Irvine, CA, USA) was performed with a Berkovich indenter under a constant loading rate of 0.2 N s^−1^. Five different indentations were conducted to ensure the mechanical reproducibility in this study. Subsize dog-bone specimens for tensile testing were machined along the rolling direction according to ASTM E8; the gauge length was 25 mm. The tensile testing was performed by INSTRON 4411 (Norwood, MA, USA) with a constant displacement rate, which yielded the initial strain rate equal to 10^−3^ s^−1^.

## 3. Results and Discussion

### 3.1. Microstructural Evolution

**Figure 1:**
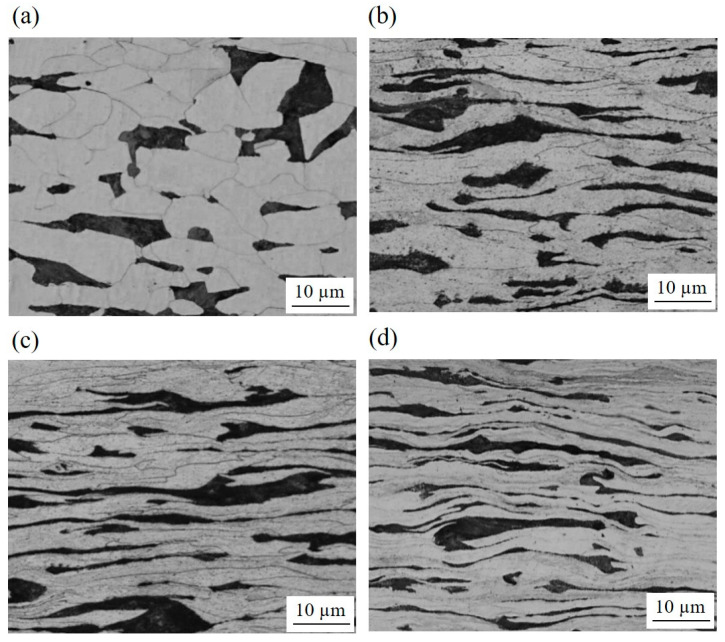
Optical micrographs of the microstructure after DSR and ESR. (**a**) After the first pass of DSR; (**b**) after the second pass of DSR; (**c**) after the fourth pass of DSR; (**d**) after the fourth pass of ESR.

Optical micrographs revealed that the grains were elongated and distorted parallel to the rolling direction (Figure 1). The distortion became apparent after the second pass. However, the structure inside the deformed grains could not be resolved by an optical microscope.

**Figure 2:**
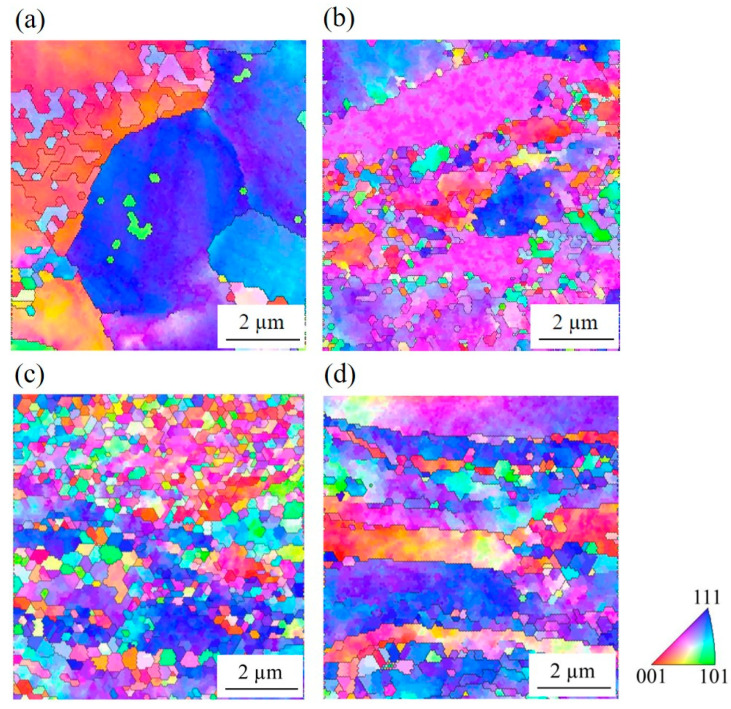
An EBSD inverse pole figure map of the microstructure after DSR and ESR. (**a**) After the first pass of DSR; (**b**) after the second pass of DSR; (**c**) after the fourth pass of DSR; (**d**) after the fourth pass of ESR. The color indicates the orientation of each grain.

EBSD observation revealed the substructure within the elongated grains (Figure 2). After the first pass of DSR, the UFG structure rarely appeared. Only after the second pass did ultrafine grains develop actively at the grain boundaries of the elongated coarse grains, which generated necklace structure. After the fourth pass of DSR, the microstructure was mostly composed of the UFG structure, with a small fraction of the elongated grains. On the other hand, the microstructure, after the fourth pass of ESR, still contained a large fraction of elongated grains, along with some ultrafine grains.

**Figure 3:**
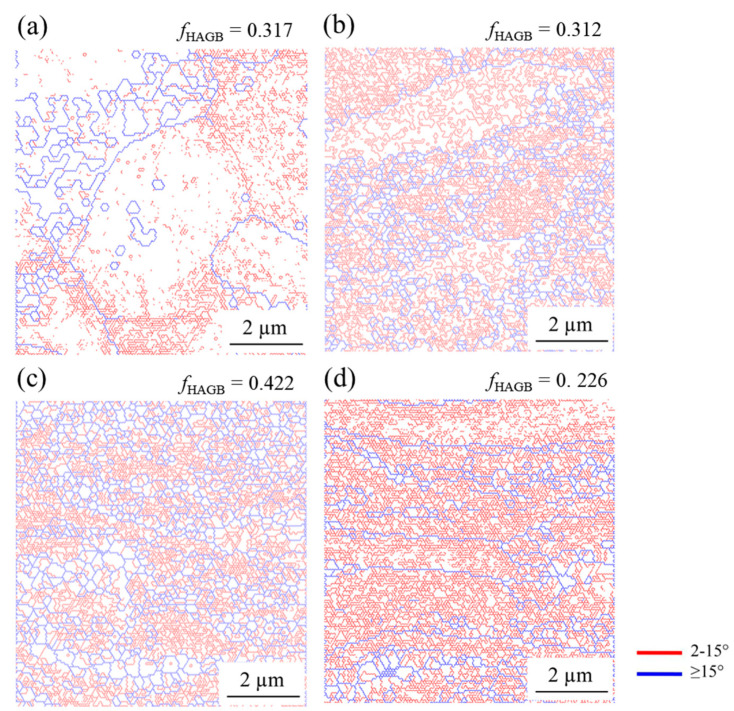
An EBSD grain boundary map of the microstructure after DSR and ESR. (**a**) After first pass of DSR; (**b**) after second pass of DSR, (**c**) after fourth pass of DSR; (**d**) after fourth pass of ESR. *f*_HAGB_ is the fraction of high angle grain boundaries.

**Figure 4:**
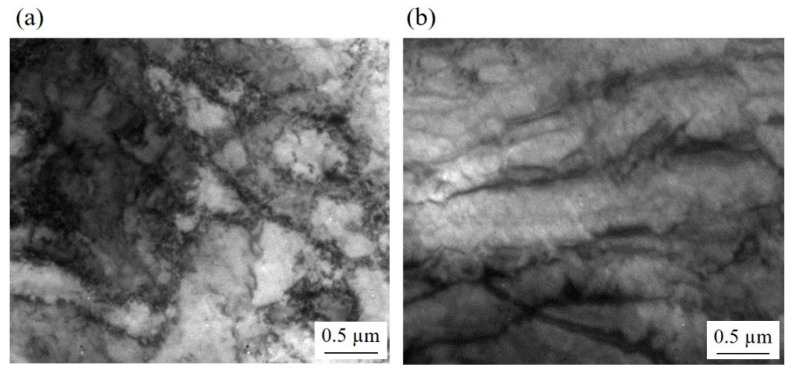
The bright field image of the microstructure after the fourth pass of (**a**) DSR and (**b**) ESR.

The microstructural difference between the samples became more pronounced when LAGB and HAGB were classified (Figure 3). The elongated grains contained a large fraction of LAGB, while the ultrafine grains were delineated by HAGB. Accordingly, the fraction of HAGB was lower in the samples after the second pass of DSR and after the fourth pass of ESR than in the alloy after the fourth pass of DSR. Consequently, the lineal intercept length of the grains surrounded by HAGB was shorter in the steel after the fourth DSR pass (0.38 ± 0.11 µm) than after the fourth ESR pass (0.65 ± 0.14 µm). Higher magnification images revealed the same trend in the deformed microstructure (Figure 4). Moreover, it was clearly observed that the grain boundaries of the UFG structure consisted of dislocations which were likely to have generated during the rolling.

The necklace structure and the alteration from elongated grains surrounded by LAGB to the UFG structure with HAGB were consistent with the microstructure formed via continuous dynamic recrystallization (cDRX). During severe plastic deformation at room temperature, polygonal grains became elongated, and a high density of dislocations were generated. The generated dislocations developed into fine cell structures whose boundaries were LAGB. As the deformation progressed, dislocations continuously accumulated at the cell boundaries. The combined Burgers vectors of these dislocations would increase the misorientation between the cells, and thereby, the boundaries gradually developed into HAGB [20,21]. Since the process occurs homogeneously throughout the material, it is referred to as “continuous”. As a result, the full UFG structure could be achieved.

### 3.2. Mechanical Behavior

**Figure 5:**
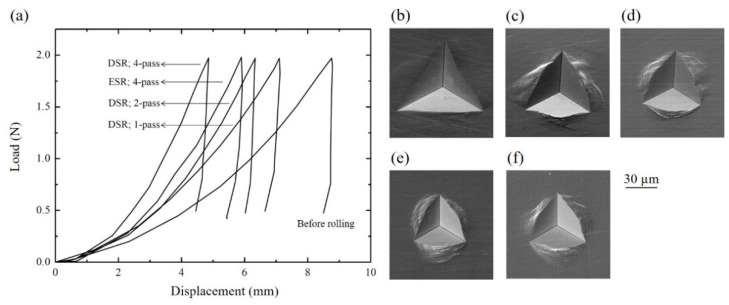
(**a**) Load-displacement curves for the samples acquired by nanoindentation. Indentation images for the samples: (**b**) prior to rolling; (**c**) after the first pass of DSR; (**d**) after the second pass of DSR; (**e**) after the fourth pass of DSR; and (**f**) after the fourth pass of ESR.

The hardness increased significantly with respect to the number of DSR passes. As the number of the DSR passes increased, the displacement at the peak load significantly decreased, and the contact area was reduced (Figure 5). As a result, the hardness effectively increased from 1.10 to 3.93 GPa after the 4 DSR passes (Table 1). Moreover, the hardness increase was more effective via DSR than via ESR. The elastic modulus did not change due to the rolling, and the values corresponded to the typical elastic modulus of steels, 208–209 GPa [22].

**Figure 6:**
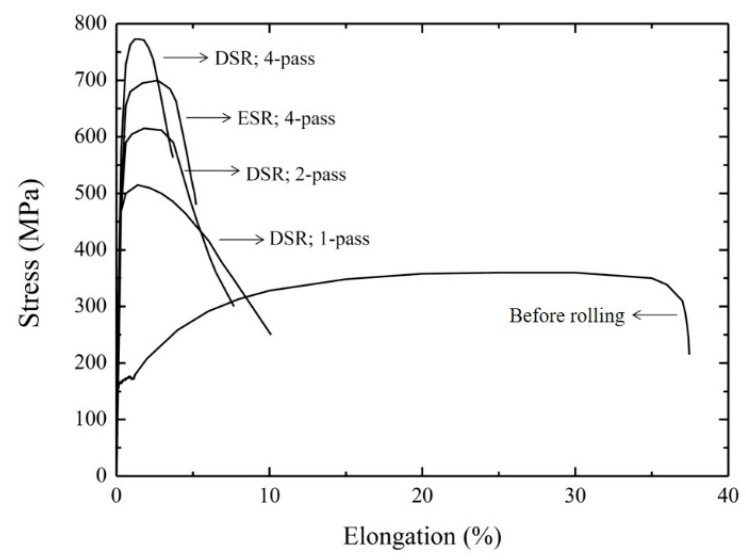
Stress-strain curves of the samples processed by rolling deformation.

As shown in Figure 6, the overall tendency of the yield strength was consistent with that of nanoindentation in that both properties increased with an increasing of the number of passes. For instance, the yield strength of the sample after a 4-pass of DSR exhibited 780 MPa, which was approximately five times higher than prior to DSR. It was noted that the yield strength after the fourth pass of ESR was lower in comparison to the case of DSR. Furthermore, the total elongation was reduced as the number of passes increased; after the fourth pass of either DSR or ESR, the total elongation was <6%.

The microstructural analysis after DSR revealed a UFG structure with a high fraction of HAGB (Figure 2 and Figure 3) and a low density of dislocations within the grains (Figure 4). Therefore, it was likely that the dramatic increase in yield strength (Figure 6) came from grain boundary strengthening, which could be explained by Hall–Petch relation;

(1) $$\Delta \sigma =k_{HP}d^{-0.5}$$

where Δ*σ* is the yield strength increment due to grain boundary strengthening, *k_HP_* is the Hall–Petch coefficient, and *d* is grain size. Gladman and Pickering stated that the yield strength of the steels which were comprised of polygonal ferrite with less than 20% of pearlite was controlled by the ferrite matrix [23]. Moreover, they suggested *k_HP_* = 15.1 MPa mm^0.5^ to explain 93% of the published data. Accordingly, the grain boundary strengthening was calculated using Equation (1) and *k_HP_* = 15.1 MPa mm^0.5^. The results (Table 2) showed that grain boundary strengthening reasonably explained the strengthening. It was noted that the acquired grain size in this work was the lower limit where the Hall–Petch relation was valid. With finer grains, the grain boundary strengthening was no longer proportional to *d*^−0.5^ and was inversely proportional to *d* [24]. The discrepancy between the actual Δσ and calculated Δσ might come from a texture which was not considered. According to the work by Huang et al., texture would influence yield strength by altering the value of the Schmid factor [25].

In addition, the low ductility of the samples produced by DSR (Figure 6) was attributed to a low strain hardening rate. The ineffective strain hardening was mainly caused by two reasons, as suggested for UFG Cu [26]; the same explanation was also provided to interpret the low total elongation of UFG Ti [27]. Firstly, only a few dislocations in each grain would contribute to deformation. Since there were a number of grains within the material, quasi-static deformation could occur via the glide of only one or two dislocations in one grain per second. Secondly, there was sufficient time for dislocations to be trapped into the non-equilibrium grain boundaries as a kind of dynamic recovery. Both reasons contributed to the absence of dislocation accumulation, and, thereby, low strain hardening rate of the samples produced by DSR.

The achieved strength level and grain size by DSR were comparable to the low carbon steels processed by ECAP (Table 3). Although the total elongation was lower after DSR than after ECAP, the yield strength was the highest among the steels with a 0.2–1.5 µm grain size. Therefore, it is suggested that DSR is a promising method for grain refinement considering its advantages over other severe plastic deformation methods regarding sample dimension and being a continuous process. Moreover, with the present data, mechanical properties or microstructures can be predicted as a future study; that is, the number of passes which result in desired material properties can be estimated.

### 3.3. Load-Stroke Behavior during DSR

**Figure 7:**
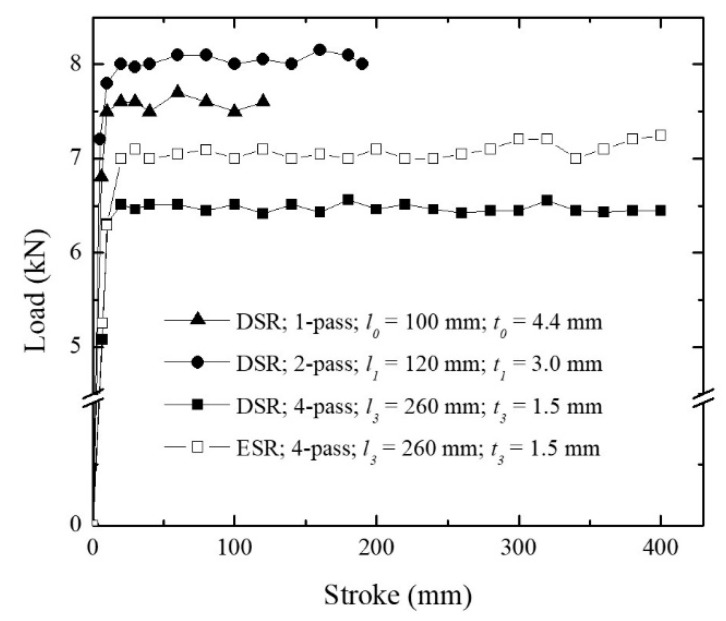
The load-stroke curves during rolling deformation.

The rolling load was mostly constant during each passage of deformation above a specific stroke point (Figure 7). However, the value did not grow monotonously as the number of passes increased. It was found that the rolling load increased during the first two passes and reached ~8.1 kN and was reduced to ~6.5 kN for the fourth pass. Moreover, the rolling load during the fourth pass of ESR was higher than that of DSR, implying that DSR required less deformation energy than ESR did. 

**Figure 8:**
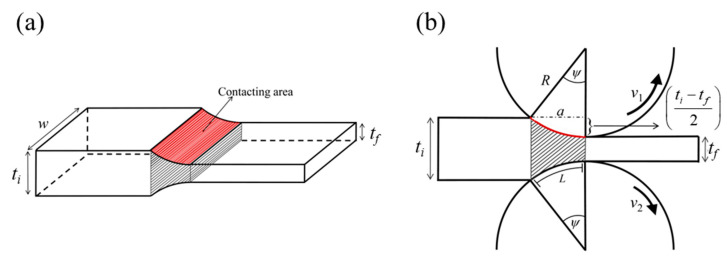
(**a**) Sample geometry and (**b**) a schematic side view during the DSR process. The contacting area and the deformation volume are shaded. *w*: sheet width; *t_i_*: initial thickness; *t_f_*: final thickness; *R*: roll radius; *ψ*: contact angle; *v*_1_ and *v*_2_: rolling velocities.

Two main factors which determine rolling load are material strength and deformation volume. The alloy strength increased as the number of passes increased (Figure 5 and Figure 6). The deformation volume can be estimated by considering the process geometry. According to Figure 8, the contacting area, *A = wa*, where *w* is the sheet width and *a* is the projected length of the contact. *a* is estimated as [32].

(2) $$a=\sqrt{R^{2}-{\left(R-\frac{t_{i}-t_{f}}{2}\right)}^{2}}=\sqrt{R\left(t_{i}-t_{f}\right)-{\left(\frac{t_{i-}t_{f}}{2}\right)}^{2}}\approx \sqrt{R\left(t_{i}-t_{f}\right)}$$

where *R* is the roll radius and *t_i_*, *t_f_* is the initial and final thickness of the process. In addition, the deformation volume *V* can be expressed as [32]

(3) $$V\approx aw\left(\frac{t_{i}+t_{f}}{2}\right)$$

*A* and *V* were calculated for different conditions, and the values are listed in Table 4. The deformation volume decreased as the number of passes increased. The variation in the material strength and deformation volume influenced the rolling load in an opposite way as the number of passes increased. In other words, with a higher number of passes, a higher load should be applied to compensate for the strain hardening of the sheets, while the deformation volume decreases and allows for the load reduction. At the first and second passes of DSR, the effect of strain hardening was higher than that of the deformation volume; therefore, the rolling load increased (Figure 7). On the other hand, after the second pass, the deformation volume decreased significantly, and its influence was dominant compared to the effect of material strength.

Interestingly, the rolling load was lower for DSR than for ESR (Figure 7). The effects of rolling speed and rolling speed ratio on the rolling force have been reported in previous studies. During the course of ESR, the increase in the rolling speed, i.e., the increase in strain rate, reduced the rolling load [33]. During DSR, it was observed that the rolling force decreased with an increase of the rolling speed ratio for both Al and Cu [34]. Moreover, it was reported that the reduction of the rolling load in an interstitial free steel could be caused not only by employing a higher rolling speed ratio but also by using lubricant, which implied that the phenomenon was related to the frictional state at the roll surface [35]. Indeed, Tzou revealed that the frictional factor decreased by increasing the roll speed ratio [36]. Consequently, higher roll speed ratio suppressed sticking friction and reduced the rolling force, resulting in homogeneous deformation. This suggests that DSR could save rolling energy via the reduction in rolling load while it attains more effective grain refinement than ESR.

## 4. Conclusions

The strengthening of a 0.18 wt % C steel via differential speed rolling (DSR) was studied and compared to its counterpart produced via equal speed rolling (ESR). The resulting microstructure, hardness, and tensile properties were investigated, along with the rolling load during the process.

1. The grains were heavily elongated along the rolling direction after the second pass. As the number of passes increased, ultrafine grains firstly formed at the grain boundaries of the elongated grains, and later spread homogeneously throughout the thickness. The fine grains were surrounded by high angle grain boundaries, which consisted of a high dislocation density. The grain refinement via DSR (*d* = 0.38 ± 0.11 μm, *f*_HAGB_ = 0.422) was more effective than via ESR (*d* = 0.65 ± 0.14 μm, *f*_HAGB_ = 0.226).
2. The strength after DSR reached 780 MPa, which was the result of an effective strengthening by high angle grain boundaries. The ductility was relatively low (~4%) for the ultrafine grain structure because dislocation accumulation was limited by the glide of dislocations and the absorption of the dislocations into the grain boundaries.
3. The rolling load increased during the first two passes up to ~8 kN due to the material strengthening. Then, the load decreased to ~6.5 kN for the fourth pass because the deformation volume was reduced. The load during DSR (~6.5 kN) was lower than during ESR (~7 kN) due to the high rolling speed ratio lowering sticking friction.

## Figures and Tables

**Table 1:** Parameters, hardness, and elastic modulus (*E*), which were acquired from the nanoindentation analysis.

Condition	hrhmax	*A* (m^2^)	Hardness (GPa)	*E* (GPa)
Before rolling	0.989	1.78 × 10^−9^	1.10	209.80
After 1-pass of DSR	0.962	1.13 × 10^−9^	1.73	210.15
After 2-pass of DSR	0.956	0.90 × 10^−9^	2.18	208.45
After 4-pass of DSR	0.929	0.50 × 10^−9^	3.93	210.18
After 4-pass of ESR	0.934	0.79 × 10^−9^	2.50	210.05

*h_r_*: residual depth of penetration, *h*_max_: maximum depth of penetration, *A*: true contact area at the maximum load.

**Table 2:** The actual increment in yield strength (Δ*σ*) and calculated Δ*σ* of the samples after the fourth pass of DSR and ESR.

Condition	*d* (μm)	Actual Δ*σ* (MPa)	Calculated Δ*σ* (MPa)
Before rolling	32 ± 4	-	-
After 4-pass of DSR	0.38 ± 0.11	600	694
After 4-pass of ESR	0.65 ± 0.14	527	508

*d*: mean lineal intercept grain size.

**Table 3:** The process parameters, grain size evolution, and tensile properties of low carbon steels produced by severe plastic deformation (SPD).

C Content (wt %)	SPD Method	Strain	Number of Passes	Deformation Temperature (K)	*d* (μm)		Tensile Properties		Reference
					Initial	Final	*σ_y_* (MPa)	*ε_tot_* (%)	
0.08	ECAP	~3	3	298	~45	~0.2	420	32	[28]
0.15	ECAP	~4	4	773	28	~0.8	581	17.6	[29]
0.15	ECAP	~4	4	773	-	~1.4	581	17.6	[30]
0.10	ECAP	~4	1	500	-	~0.3	650	35	[31]
0.18	DSR	~7.6	4	298	32	0.4	780	4	Present study

*d*: mean lineal intercept grain size, *σ_y_*: yield strength, *ε_tot_*: total elongation.

**Table 4:** The calculated contacting area (*A*) and deformation volume (*V*). For relative values, the values of 1-pass of DSR were set as reference.

Condition	*A*		*V*	
	Absolute (mm^2^)	Relative	Absolute (mm^3^)	Relative
1-pass of DSR	372	1.00	1392	1.00
2-pass of DSR	314	0.84	822	0.59
4-pass of DSR	199	0.63	253	0.30

## Data Availability

The data presented in this study are available on request from the corresponding author. The data are not publicly available because they are part of an ongoing study.
